# Virtual Reality Exposure Therapy for Fear of Heights: Clinicians’ Attitudes Become More Positive After Trying VRET

**DOI:** 10.3389/fpsyg.2021.671871

**Published:** 2021-07-15

**Authors:** Elise Rimer, Lars Vågsholm Husby, Stian Solem

**Affiliations:** Department of Psychology, Norwegian University of Science and Technology, Trondheim, Norway

**Keywords:** virtual reality, VR, exposure therapy, attitudes, fear of heights, acrophobia

## Abstract

**Background:**

Virtual reality exposure therapy (VRET) has the potential to solve logistic challenges when treating specific phobias. However, VRET has yet to see a large-scale implementation in clinical settings despite positive findings in treatment trials. This may partly be due to attitudes and lack of experience among clinicians, but also because of expensive and stationary VR solutions.

**Objective:**

This study tested whether modern, wireless, commercially available VR equipment with controller-free hand tracking could induce and reduce discomfort using scenarios designed for fear of heights. Also, the study tested if clinicians’ attitudes toward using VR in therapy changed after trying it themselves.

**Method:**

Attitudes to using VR in therapy and discomfort ratings were assessed for 74 clinicians before and after completing two VR scenarios. In addition, 54 non-clinicians completed the same scenarios. Participants were not diagnosed with acrophobia.

**Results:**

The VR scenarios induced discomfort comparable to participants’ reported fear of heights in real life. Repeated training reduced discomfort. Positive attitudes toward use of VR in therapy was predicted by previous experience with VR, as well as positive attitudes toward novel technology and exposure therapy. Clinicians’ attitudes became more favorable after trying VRET themselves. Clinicians reported a range of possible advantages and disadvantages of using VR in therapy.

**Conclusion:**

VRET for fear of heights was able to induce and reduce discomfort in clinicians and non-clinicians, and clinicians’ attitudes toward using VRET become more positive after trying VRET for themselves. The latest generation of VR solutions has potential to improve clinical availability and treatment options. Future research should explore how VRET can be implemented in clinical settings.

## Introduction

About one third of the population suffers from susceptibility to acrophobia (fear of heights) and visual height intolerance (Huppert et al., 2020). For the purposes of this study, fear of heights is regarded as an inter- and intra-individual continuum of varying degrees of discomfort that may be elicited by visual heights. Research suggests that exposure therapy is the most robust treatment available for specific phobias (Deacon and Abramowitz, 2004). On the other hand, exposure therapy is not without its limitations. Issues with confidentiality can arise when treatment takes place outside the therapy room, and it can be difficult to access the feared stimuli (Anderson et al., 2004).

Although the treatment efficacy of exposure therapy is well established, many cases remain untreated or even undiagnosed (Neudeck and Einsle, 2012). In a study by Hipol and Deacon (2013), they discovered that only 19–33% of patients treated for anxiety disorders received *in vivo* exposure. They pointed to different reasons for this: (1) Structural challenges like time, space, and logistics, (2) clinicians’ attitudes toward exposure techniques, (3) reservations among clinicians, and (4) lack of knowledge and skills concerning how to apply exposure appropriately. This calls for more practical means of delivering exposure-based treatments, efforts to challenge attitudes and reservations among clinicians, and opportunities for clinicians to gain experience.

The growing body of research on virtual reality exposure therapy (VRET) shows promising results, particularly for specific phobias (Parsons and Rizzo, 2008; Powers and Emmelkamp, 2008; Opriş et al., 2012; Morina et al., 2015; Botella et al., 2017; Lindner et al., 2017; Carl et al., 2019). VRET may offer some advantages as opposed to *in vivo* exposure therapy, such as being more appealing to some patients, more cost-effective, afford possibilities for more gradual exposure (Emmelkamp, 2005), as well as mitigating structural barriers like time and logistics (Neudeck and Einsle, 2012; Bouchard et al., 2017). In addition, there are indications that drop-out rates are lower with VR treatments and that patients could have a preference for VR exposure over *in vivo* treatment (Garcia-Palacios et al., 2001, 2007). Furthermore, a meta-analysis proposed that the deterioration rate in VRET is low (4%), and comparable with face-to-face therapy (Fernández-Álvarez et al., 2019).

Another potential advantage of VR is that patients know that the virtual environment is not real, but their minds and bodies respond as if it is; which could explain why VRET may offer improvements that are generalizable to the real world (Freeman et al., 2017). VR also affords the possibility of various scenarios and stimuli tailored to individual needs, reduced inconsistency of treatment delivery, as well as eliminating the need for real-world stimuli that might be difficult to procure and/or manipulate in a therapeutic context (Bouchard et al., 2017; Freeman et al., 2017; Boeldt et al., 2019). In theory, any real situation can be simulated in VR, and the virtual environment can be extended by elements that would not be feasible in the real world.

However, potential limitations of VRET need to be considered. Considering the constant upgrades of technology, there is a scarcity of studies utilizing newer hardware. Earlier generations of VR products are usually wired, which can hinder mobility, and consequently immersion by restricting free movement in the VR simulation. Immersion is important to consider as it might be a prerequisite for fear responses (Price and Anderson, 2007; Gromer et al., 2018), but anxiety could also increase the feeling of presence (Bouchard et al., 2008). Additionally, products that utilize handheld controllers might be unintuitive for some individuals. Although some research has been done with newer hardware, there is no evidence to suggest that recently developed VR products – with better resolution, mobility, and controller-free hand tracking – are more clinically successful than older systems (Jerdan et al., 2018). However, immersive capabilities are likely to be better with newer VR systems. Studies also indicate that the potential of cybersickness (i.e., nausea, headaches, and/or dizziness as a side effect of VR use) can be a considerable barrier for some patients (Weech et al., 2019), and this has been a challenge with older generations of VR headsets (Rebenitsch and Owen, 2016).

Despite a growing number of novel technological inventions, we have yet to see widespread implementation of the technology into clinical settings (Gega, 2017; Mishkind et al., 2017). This may partly be due to lack of acceptance and technological literacy among clinicians (Anton and Jones, 2017). However, a study where eight clinicians were interviewed after trying VR seemed to indicate willingness and potential for implementation (Ose et al., 2019). Furthermore, many clinicians perceive exposure therapy to be unethical, harmful, and intolerable (Gunter and Whittal, 2010; Deacon and Farrell, 2013). However, studies also indicate that negative attitudes toward exposure therapy can be changed through training and experience (Deacon et al., 2013). Thus, it seems plausible that negative attitudes toward VRET might change through training and experience as well.

A recent cross-sectional survey of cognitive-behavioral therapists found significantly higher average positive scores than average negative scores regarding their views on VRET, but negative attitude was a larger predictor of future use (Lindner et al., 2019). They concluded that therapists’ attitudes did not seem to constitute a major barrier to implementation of VR in clinical practice, and that CBT clinicians overall had positive attitudes toward VRET. Commonly held negative concerns were: low realism; that therapy will not translate into real-world improvements; and the possibility of technical difficulties. Technological and organizational factors, such as major expenses, technological complexity and low graphical fidelity, have also been mentioned as barriers to implementation of VR as a therapeutic tool in psychiatric treatment (Ramsey et al., 2016; Slater and Sanchez-Vives, 2016; Glegg and Levac, 2018).

As technological barriers dissipate, it is of fundamental interest to investigate predictors of clinicians’ general attitude toward the use of VR in therapy, and document changes in attitudes after trying a VRET program. In addition, the therapeutic value of the specific VR software and hardware needs to be established by empirically testing its ability to induce discomfort at different levels of intensity. Finally, clinicians’ views of the pros and cons of VR need to be further examined. These are the aims of this study.

This study utilizes VR hardware with certain features that, to our knowledge, have not been used in previous studies on VRET. The VRET product in this study is wireless and utilizes controller-free hand tracking, which allows the user to move more freely – thereby also sustaining immersion – and affords multisensory integration, which could be clinically beneficial (Wiederhold and Riva, 2019). Fully tracked hands and articulated fingers can potentially enhance engagement and deliver a more natural interaction with the environment (Park et al., 2019).

This study tested the following hypotheses:

1. The VR intervention will induce Subjective Units of Discomfort (SUD) ratings comparable to the participants’ experience of heights in real life. Furthermore, repeated exposure to the height scenarios will reduce SUD ratings in this undiagnosed sample of clinicians and non-clinicians.
2. Young age, male sex, few years of experience as a clinician, previous experience with VR and VRET, favorable attitudes toward novel technology and exposure therapy, and high technological literacy will predict a more favorable general attitude toward the use of VR in therapy among clinicians.
3. Clinicians’ general attitude toward use of VR in therapy, how supplemental VR seems in therapy, and an assessment of the feasibility and usefulness of VR in therapy will be rated higher after trying VRET.
4. Clinicians can see pros and cons of using VR in therapy.

## Materials and Methods

### Participants and Procedure

The sample was partly obtained by email invitations to employees at a University psychology department, and private and 10 public health clinics across Norway. In total, clinicians from nine different clinics were recruited. In addition, fifth and sixth year psychology students (with clinical experience) and psychologists were recruited using Facebook invitations. We also recruited 54 non-clinicians using convenience sampling (participants were asked to share information about the project to friends, family, and colleagues).

All willing adult respondents were permitted to participate with no exclusion based on their degree of fear of heights. The sample was divided between clinicians (*n* = 74) and non-clinicians (*n* = 54). The final sample consisted of 128 consenting adults (83 women and 45 men), with age ranging from 18 to 70 years (*M* = 34.64, SD = 12.33).

The clinician sample consisted of 28 clinical psychology graduate students with clinical experience, 24 therapists from a local psychiatric clinic, 6 from the university, 6 from a child and adolescent outpatient clinic, 4 from primary health care services, 4 from a substance abuse clinic, 1 from a habilitation clinic, and 1 from an inpatient clinic. The clinicians had 7.16 (9.93) years of experience (range 0–38). Twenty-five percent of the clinicians used exposure therapy in their work, and those who did had 6.81 (5.95) years of experience with doing so (range 0–20). The clinicians also reported a markedly favorable attitude toward exposure therapy with a mean score of 4.68 (0.55) on a 1–5 scale. Forty-five percent of the clinicians had tried VR before, while 3.9% had tried VRET.

### Measurements

The self-report questionnaires were composed of the following sections: demographics, therapeutic background, subjective discomfort during the interventions, immersion, altitude perception, and technological literacy. Moreover, certain attitudes (i.e., general attitude toward VR in therapy, exposure therapy, and novel technology) were measured, as well as the perceptions of benefits and cost of VR in anxiety/phobia treatment, the degree of which VR can be a supplemental tool in anxiety/phobia treatment, and the feasibility of anxiety/phobia treatment with VR therapy. A six-point Likert scale was chosen to compel respondents to take a position, by giving them no option to take a neutral stance. Attitudes were scored on six-point Likert scales ranging from (0) *extremely negative*, (1) *very negative*, (2) *quite negative*, (3) *quite positive*, (4), *very positive*, (5) *extremely positive*. Clinicians’ pre-intervention assessment of the utility of VR in therapy, their own technological literacy, and post-intervention ratings were scored on a six-point Likert scale ranging from (0) *not at all*, (1) *to a small extent*, (2) *to some extent*, (3) *to a moderate extent*, (4) *to a great extent*, (5) *to a very great extent*. Clinicians were also asked to fill out empty text boxes with suggested advantages and disadvantages of using VR in therapy. Non-clinicians were given a simplified questionnaire excluding variables concerning therapy and technological literacy. The questionnaires are included in the Supplementary Appendix.

Levels of discomfort were measured using a SUD Scale. The participants were asked to rate their level of situational discomfort from 0 (i.e., *no discomfort*) to 100 (i.e., *extreme discomfort*) at five different time points: (1) Before the VR intervention, a suggested maximum level of discomfort when hypothetically faced with extreme heights in real life was measured, (2) Additionally, all participants reported an *in vivo* reference to their fear of heights while standing on a footstool (50 cm in height with a surface area of 39 cm × 25 cm). In total, three SUDs ratings were reported during the VR interventions and written down by the test administrators. The first and second of these were measured at peak discomfort in both VR scenarios (see below for details). The third was measured at the end of the second scenario, approximately 2 min after peak discomfort.

### Interventions

The VRET software was developed by Fornix in Trondheim. The program utilized the VR hardware Oculus Quest, which is a wireless head-mounted display (HMD) with internal processing power, and consequently does not require a separate computer to be functional. It included a controller-free hand tracking feature, which enabled the use of hands as an input method to control the program. The HMD also delivered simulated surround sound, which was audible to both the participant and test administrator. This feature is clinically relevant considering the potential for increased discomfort when visual stimuli is combined with auditory stimuli (Taffou et al., 2013). Furthermore, the HMD had a low input lag, which can potentially mitigate loss of immersion and cybersickness (Kim et al., 2020). The software consisted of two different scenarios, which will be referred to as the “Lift” and the “Plank.” The scenarios required an unobstructed movement area of 2 × 3 m. Videos demonstrating the scenarios can be found at fornixvr.com/our-solutions and youtube.com/watch?v=p04vMpX4_MI&t=39s.

**FIGURE 1:**
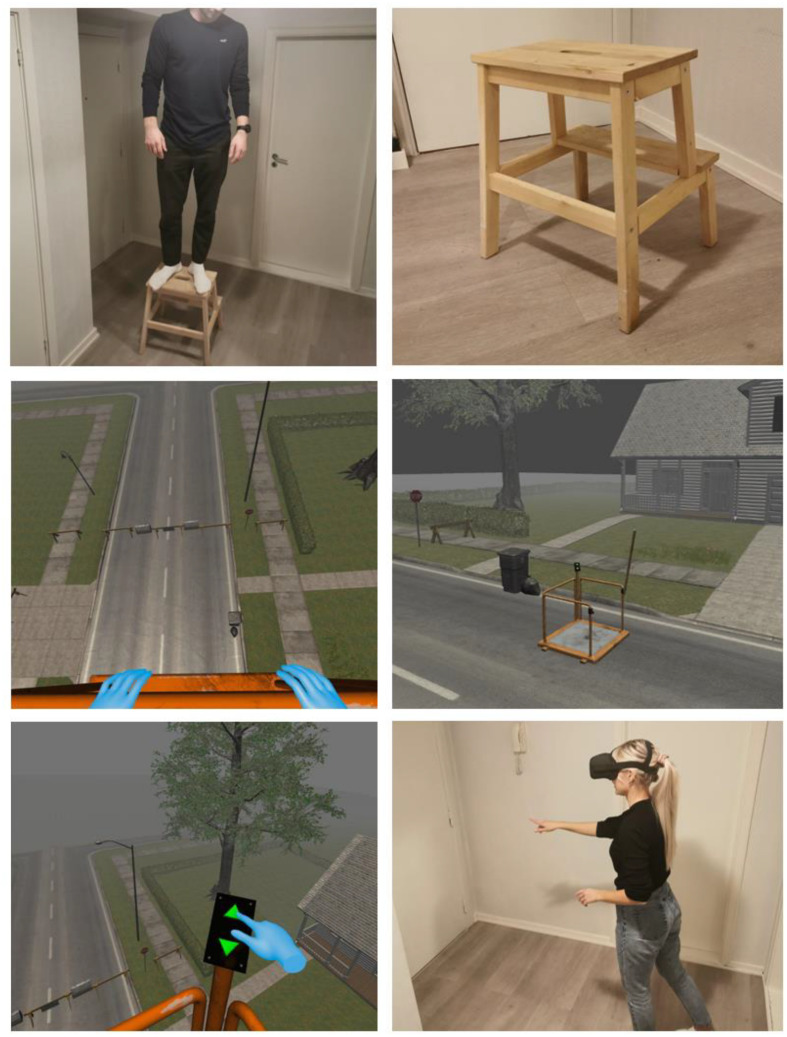
Inner and outer perspectives of the lift-intervention.

**FIGURE 2:**
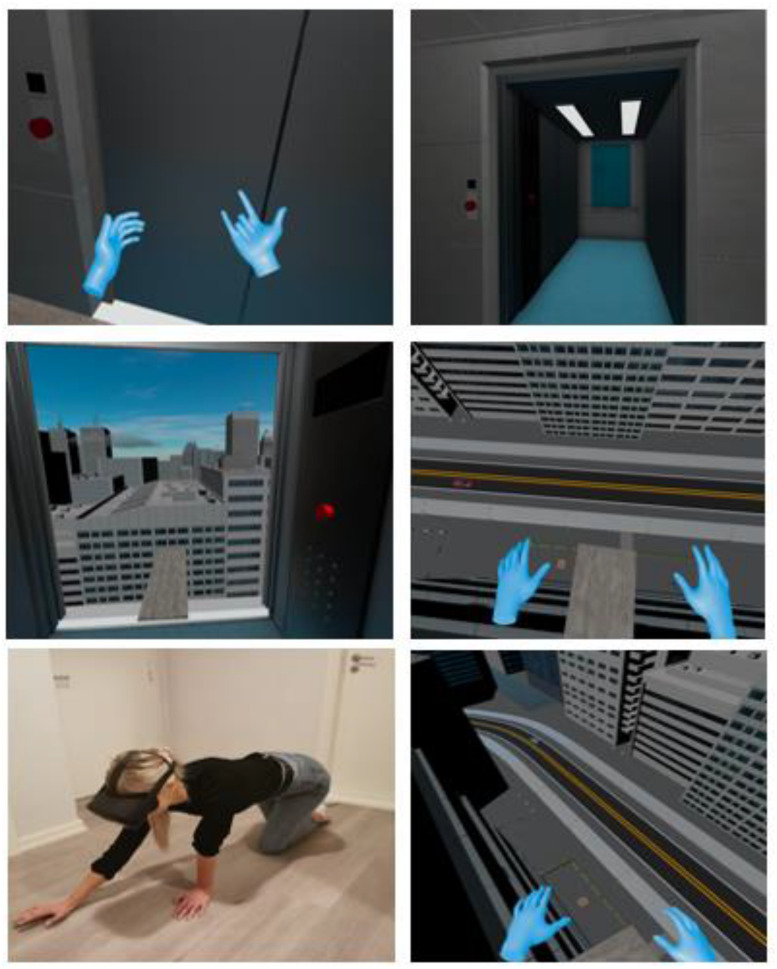
Inner and outer perspectives of the plank-intervention.

All participants were initially asked to climb a footstool and rate their subsequent discomfort from 0 to 100. After this, they were instructed to wear the HMD to experience the VR scenarios. Brief verbal instructions were given to inform participants how to navigate the environment (e.g., moving around and pressing buttons). The Lift simulated a 3D environment where the participants entered a lift that could be elevated to six different levels of altitude (see Figure 1). It had a low railing and was located on a street surrounded by houses and trees. In addition, the audio consisted of the sound of the wind, the rustling of branches, bird calls, the interactive console buttons, and the lift mechanics moving up and down. The participants were always in control and could choose how high they wanted the lift to go by pushing the arrow-button upwards or downwards. The lift elevated in non-linear increments (i.e., 1.5, 2, 3, 4, 5, and 6 m above ground). The top level was at an elevation of 21.5 m. To advance to the next intervention, the participants formed a special hand-gesture (see top-left illustration in Figure 2) which transported them almost instantaneously to the Plank-scenario.

In the Plank-scenario, the participants were initially standing outside of an elevator (see Figure 2). The door was opened by reaching out a hand to the left of the elevator and pressing the red button. The participants then walked inside and pressed another red button, which caused the elevator to go up. The elevator door opened at the 20th floor of a tall building, approximately 60 m above ground (without incremental elevation). The participants were then presented with a narrow plank without railings and were encouraged to move out onto the plank at their own pace. After reaching their peak discomfort, the participants were encouraged to stay in the virtual environment for 2 min or more. The audio consisted of the sound of button pressed, the door opening/closing, and music while the elevator was moving. While on the plank, the audio consisted of strong winds.

Participants were guided through both scenarios while simultaneously rating their subjective discomfort. Various strategies were implemented by the test administrators to induce discomfort. The strategies utilized were mainly the same between participants, but some variation was necessary as it was contingent on the individual participants’ willingness to engage in the given challenges. Several participants were encouraged to jump up and down or move off the lift/plank to see if the discomfort would increase when their feet were not firmly planted to the ground. After experiencing “walking on air,” participants were asked to move back to the plank and remain there for a short while to see if their anxiety would subside. Some of the more anxiety prone participants were not inclined to walk off the lift or plank and were instead asked to visualize moving off the plank or the lift or just to feel the “air” with their foot or their hands. If the height experience became too overwhelming, participants were encouraged to take their time, breathe, or feel the wall or the floor. Some felt it safer to crawl out on the plank, others preferred to walk fast, while a few did not want to walk on to the plank at all. Most of the participants moved carefully onto the plank and were eventually able to comply with any given challenge. None of the participants were forced to do anything they did not want to do, but all were encouraged to try.

### Data Analyses

The first hypothesis (VRET will induce SUD ratings comparable to participants’ experience of heights in real life) and the sub-hypothesis (repeated exposure to the height scenarios will reduce SUD ratings) was tested using repeated measures ANOVA. The dependent variable was the SUD ratings and the ANOVA was repeated four times in chronological order of the interventions, specifically: Footstool, Lift (peak), Plank (peak), and Plank (end). The effect of time across the interventions was tested using Wilks’ lambda and pairwise comparisons. The second hypothesis (young age, male sex, few years of experience as a clinician, previous experience with VR and VRET, favorable attitudes toward novel technology and exposure therapy, and high technological literacy will predict a favorable general attitude toward use of VR in therapy) was tested using regression and correlation analyses. The third hypothesis (attitudes toward VR in therapy will become more favorable after the VR intervention) was tested using a paired *T*-test comparing pre-intervention scores with post-intervention scores. Change in mean scores following the intervention was also evaluated using Cohen’s *d* effect size using pooled standard deviations. The fourth hypothesis (clinicians can see pros and cons of using VR in therapy) was explored by categorizing and counting responses provided by the clinicians.

For the repeated measures ANOVA, 12 participants were excluded due to missing SUD ratings. For the correlation and regression analyses there was one missing value. Missing data was not replaced. The study protocol was approved by the Norwegian Centre for Research Data (NSD), reference number: 386880. Additionally, the protocol was approved by the Regional Committee for Medical and Health Research Ethics (REK; reference number: 100564).

## Results

Comparisons between non-clinicians and clinicians are presented in Table 1. Non-clinicians had a significantly more favorable general attitude toward novel technology than clinicians (*d* = 0.61). Other differences between the subsamples were insignificant. Moreover, mean ratings of general attitude toward novel technology, immersion, and altitude perception were notably high for both subsamples (see Table 1). Some dizziness (i.e., cybersickness) were reported by three participants (2.3%).

**TABLE 1:** Comparisons of clinician (*n* = 74) and non-clinician (*n* = 54) samples.

	**Clinicians**	**Non-clinicians**			
		
	***M* (SD)/% (*n*)**		***t*/*x*^2^**	***p***	***d***
1. Age	34.64 (11.85)	34.63 (13.07)	−0.00	0.998	
2. Female sex	66.22%(49)	62.96%(34)	0.15	0.703	
3. Attitude novel technology	4.12 (0.70)	4.52 (0.61)	3.35	0.001	0.61
4. Immersion	4.00 (0.99)	4.19 (0.75)	1.15	0.253	
5. Altitude perception	4.22 (0.83)	4.35 (0.73)	0.96	0.340	

*Variable 3 was scored on a scale from 0 (extremely negative) to 5 (extremely positive). Variables 4 and 5 were scored on a scale from 0 (not at all) to 5 (to a very great extent).*

### Did the VR Interventions Induce and Reduce SUD?

**FIGURE 3:**
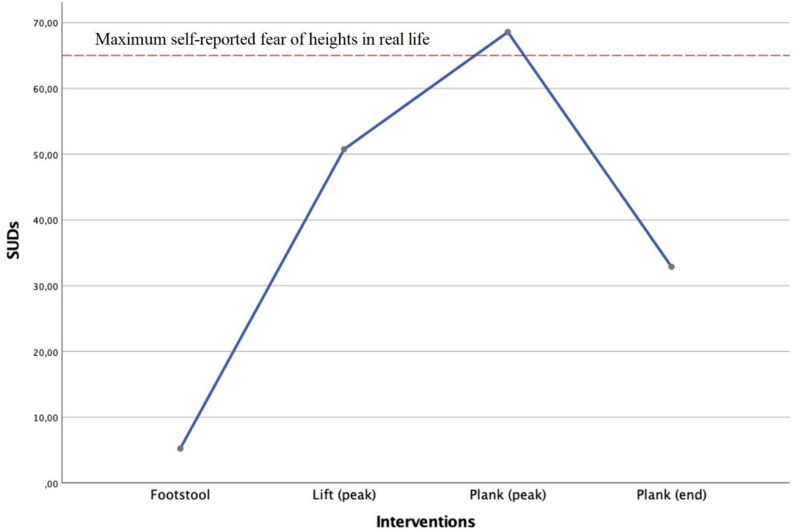
Visualization of SUD ratings throughout the interventions for all participants.

There was a significant effect of the interventions, Wilks’ Lambda = 0.15, *F*(3, 113) = 215.13, *p* < 0.001. SUD ratings were significantly higher in the Lift (peak)-intervention (*M* = 50.73, SD = 29.64) compared to the Footstool-intervention (*M* = 5.23, SD = 11.53), *t*(115) = −16.95, *p* < 0.001; *d* = 2.02. Furthermore, SUD ratings were significantly higher in the Plank (peak)-intervention (*M* = 68.57, SD = 28.66) compared to the Lift (peak)-intervention, *t*(115) = −7.57, *p* < 0.001; *d* = 0.61. Finally, SUD ratings were significantly lower in the Plank (end)-intervention (*M* = 32.87, SD = 29.19) compared to the Plank (peak)-intervention, *t*(115) = 15.78, *p* < 0.001; *d* = 1.23. It is also worth noting that the highest level of mean SUD ratings surpassed the mean suggested maximum fear of heights in real life (*M* = 64.99, SD = 28.07; see Figure 3).

### What Factors Are Associated With Positive Attitudes Toward VR in Therapy?

Correlations between the study variables are presented in Table 2. General attitude toward use of VR in therapy was significantly correlated general attitude toward novel technology (*r* = 0.37), and general attitude toward exposure therapy (*r* = 0.28). Men had higher scores than women on general attitude toward novel technology (*t* = −2.50, *p* = 0.015) and technological literacy (*t* = −3.93, *p* < 0.001). Furthermore, clinicians that had tried VR before had a more favorable attitude toward using VR in therapy (*t* = 2.18, *p* = 0.033).

**TABLE 2:** Pearson’s correlation coefficients of the study variables for the clinician sample.

	**1**	**2**	**3**	**4**	**5**	**6**
1. Att. VR therapy	–					
2. Age	−0.08	–				
3. Clinical exp.	−0.07	0.94**	–			
4. Att. novel tech.	0.37**	0.15	0.13	–		
5. Attitude ET	0.28*	−0.14	−0.15	0.17	–	
6. Tech. literacy	0.14	−0.10	−0.13	0.31**	0.11	–

VR, virtual reality; VRET, virtual reality exposure therapy; Att. VR therapy, general attitude toward use of VR in therapy; Att. novel tech., general attitude toward novel technology; ET, exposure therapy; Tech. literacy, technological literacy.

*p < 0.05. **p < 0.01.

An overview of a multiple linear regression predicting general attitudes toward use of VR in therapy (pre-intervention) is presented in Table 3. The explanatory variables in the regression analysis were sex, age, years of experience as a clinician, previous (non-clinical) experience with VR, previous experience with VRET, general attitude toward novel technology, general attitude toward exposure therapy, and technological literacy. Results of the multiple linear regression indicated that there was a collective significant effect between the aforementioned explanatory variables, *F*(8, 64) = 3.36, *p* = 0.003, *R*^2^_Adjusted_ = 0.21. Variables associated with a positive general attitude to use of VR in therapy were previous (non-clinical) experience with VR, a positive general attitude toward novel technology, and a positive general attitude toward exposure therapy.

**TABLE 3:** Predictors of general attitude to use of VR in therapy in the clinician sample.

	**β**	***t***	***p***
Sex	−0.12	−0.90	0.371
Age	−0.31	−0.97	0.334
Clinical experience	0.31	0.93	0.355
Tried VR	0.36	3.04	0.003
Tried VRET	−0.17	−1.35	0.183
Attitude novel technology	0.42	3.52	0.001
Attitude exposure therapy	0.23	2.09	0.041
Technological literacy	−0.02	−0.15	0.882

*VR, virtual reality; VRET, virtual reality exposure therapy; Sex, 0 = female, 1 = male; Clinical Experience, is the number of years as a clinician; Tried VR, 0 = no, 1 = yes.*

### Do Attitudes Toward VR in Therapy Become More Favorable After Trying VRET?

Table 4 presents changes in attitudes pre-intervention and post-intervention. This analysis pertains exclusively to clinicians. General attitude toward use of VR in therapy was significantly more favorable post-intervention compared to pre-intervention (*d* = 0.86). Next, the usefulness of VR in therapy was rated significantly higher post-intervention compared to pre-intervention (*d* = 0.89). Furthermore, VR as a supplemental tool in therapy was also rated significantly higher post-intervention compared to pre-intervention (*d* = 0.46). Lastly, the feasibility of VR in therapy was also rated significantly higher post-intervention compared to pre-intervention (*d* = 0.70). All of these changes can be considered large effect sizes, with the exception of the supplemental nature of VR in therapy, which corresponds to a medium effect size. The ratings were markedly high both pre- and post-intervention.

**TABLE 4:** Clinicians’ attitudes to using VRET pre- and post-intervention (*n* = 74).

	**Pre**	**Post**	***t***	***p***	***d***
1. General	3.92 (0.74)	4.49 (0.58)	−6.75	<0.001	0.86
2. Useful	3.99 (0.65)	4.54 (0.58)	−6.76	<0.001	0.89
3. Supplemental	4.35 (0.65)	4.64 (0.59)	−3.74	<0.001	0.46
4. Feasible	4.23 (0.73)	4.69 (0.57)	−5.76	<0.001	0.70

*Variables were scored on a 0–5 scale.*

### What Are the Perceived Pros and Cons of VR in Therapy?

**FIGURE 4:**
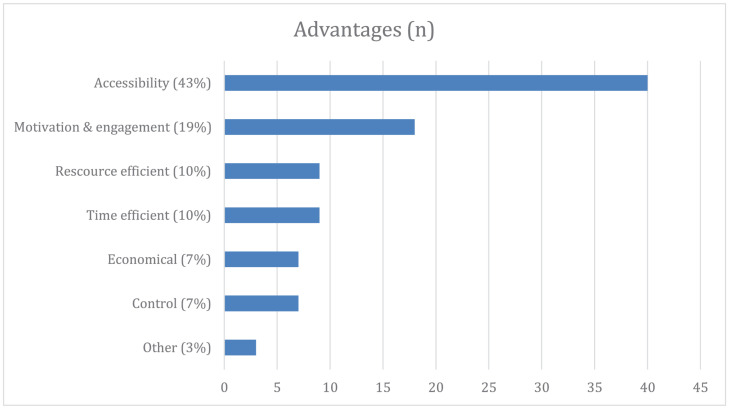
Clinicians’ perceived advantages of using VR in therapy before the intervention. Accessibility, easy implementation and accessibility of different scenarios and stimuli without having to move out of the therapist office; Motivational and engaging, lower dropout rates, more engaging for youth and adolescence, gamification; Resource efficient, can be done solely by the therapist with no need to procure *in vivo* anxiety evoking stimuli; Time efficient, saving time by not having to seek out anxiety evoking stimuli. More time in therapy and less time spent on arranging exposure experiments; Economical, no expenses regarding *in vivo* exposure stimuli (e.g., airplane tickets, dental appointments, etc.) and reduced time related expenses; Control, all variables can be manipulated by clinicians and a greater degree of patient agency (e.g., the HMD can be removed at any time); Other, a residual category for items with low frequency of occurrence; relaxation training (*n* = 1), between session training (*n* = 3), standardized approaches (*n* = 2).

**FIGURE 5:**
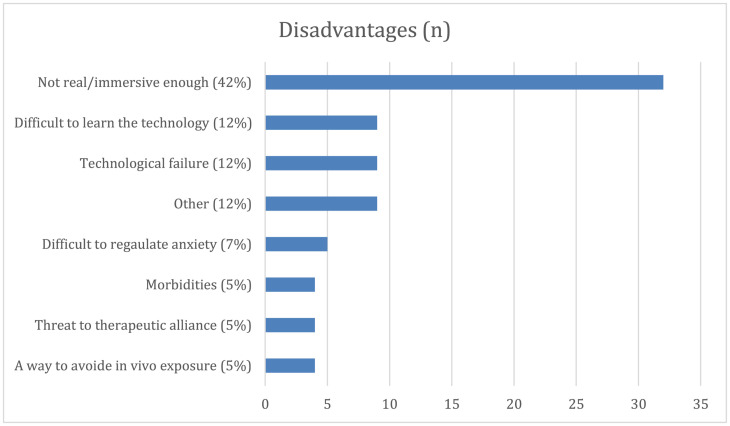
Clinicians’ perceived disadvantages of using VR in therapy before the intervention. Not real/immersive enough, low degree of realism and generalizability to real-world settings; Difficult to learn the technology, worries that it will be difficult learning to operate the equipment; Technological failure, worries that the equipment could malfunction; Other, a residual category for items with low frequency of occurrence; high cost of equipment (*n* = 3), cybersickness (*n* = 3), not more effective than regular *in vivo* treatment (*n* = 1), not enough research on VRET (*n* = 1), few patients with specific phobias are treated at general outpatient clinics (*n* = 1); Difficult to regulate anxiety, no easy way to control the degree of exposure; Morbidity, unfit to use on people with diagnoses like psychosis or other limitations like impaired hearing and/or vision; Threat to therapeutic alliance, potential barrier between clinician and client; A way to avoid *in vivo* exposure, allowing the patient to avoid exposure to real-world stimuli.

In the pre-intervention questionnaire, the clinicians reported suggestions of benefits and drawbacks of using VR in therapy. The questions were open ended, and participants were asked to elaborate. The majority gave detailed accounts of perceived advantages and disadvantages, but eight respondents did not report advantages and 20 respondents did not report disadvantages. Figures 4, 5 illustrate a summary of their answers. The most frequently reported advantages of using VR in therapy included accessibility to different objects, situations, and tasks that can only be done in a virtual setting as well as always having exposure material at hand. Furthermore, factors relating to motivation and engagement were frequently mentioned. Other frequently emphasized advantages were resource and time efficiency, and the ability to control and tailor the exposure sessions to individual needs.

Concerns that the program and VR environment would not be experienced as real enough was the most frequent suggested disadvantage. Difficulties in learning how to use the technology and the possibility of technical failure were also frequently mentioned.

## Discussion

The VRET interventions designed for fear of heights induced discomfort in the participants. On account of verbal reports during the interventions and the measures of immersion and altitude perception, we interpret the results as evidence that the reported discomfort is due to the participants’ varying degrees of fear of heights. Taking into account that SUD ratings exceeded their self-reported maximum discomfort of heights in real life, this suggests that VR is able to simulate phobic stimuli to induce discomfort in this undiagnosed sample, comparably to real-world stimuli. Considering the tenable need for accessible stimuli in exposure therapy, this demonstrates the value of VR as a therapeutic tool. Additionally, we found a decrease in levels of discomfort when participants were given a short period of time to desensitize to the anxiety inducing situation in VR. This finding is in line with studies on VRET using clinical samples (Carl et al., 2019).

Also, in accordance with previous research by Lindner et al. (2019) and Segal et al. (2011), we found that previous experience with VR, a favorable attitude toward novel technology, and attitude toward exposure therapy predicted favorable general attitude to the use of VR in therapy. We did not find support for an effect of sex or age, which seems to verify the findings of Segal et al. (2011). Therefore, it seems that VR might have broad appeal among clinicians. The increasingly positive attitudes after experiencing VRET is in line with prior research establishing a link between VR experience and attitudes (Ose et al., 2019). As reported by Lindner et al. (2019) and Ose et al. (2019), we also found that clinicians have an overall positive attitude toward VR in therapy. Moreover, this pre-intervention positivity did not prevent clinicians’ attitudes to become significantly more favorable post-intervention. This initial positivity could partially be due to a sampling bias.

The clinicians’ perceived advantages of using VR in therapy seem to be in line with the findings of Lindner et al. (2019) and Segal et al. (2011). These include the benefits of high accessibility to different objects, situations, and tasks; motivation and engagement; resource and time efficiency; and tailored experiences with increased levels of patients’ and therapist control, as well as agency. In light of the distinctly positive attitudes of our sample, these considerations are presumably representative of the thoughts of positively inclined clinicians.

Additionally, perceived disadvantages are also in accordance with Lindner et al. (2019) and Segal et al. (2011), which similarly include concerns regarding technical failure, difficulties in learning the technology, and low realism/immersion with therapeutic improvements not being generalizable to real-world settings. Despite the concerns regarding graphic fidelity, immersion, and *in vivo* generalizability, there is negligible empirical support for these suggested downsides (Ling et al., 2014; Morina et al., 2015). Technological barriers such as issues with technical difficulties, low graphic fidelity, cybersickness, and expenses, are becoming increasingly inconsequential with the wider availability of consumer VR products, but human barriers still prevail.

Importantly, as demonstrated by Lindner et al. (2019) and Segal et al. (2011), the existence of negative attitudes is likely to be a bigger obstacle to the implementation of VR in clinical settings than the absence of positive attitudes. Consequently, negative concerns seem to be primary targets that need to be addressed in order for a large-scale implementation of VRET to take place. Our study highlights similar negative concerns and solidifies the potential targets in efforts to facilitate implementation of VR in clinical settings. Some concerns may be alleviated through training programs, with validated protocols, but it seems to be just as important to let clinicians experience the new VR technology for themselves.

The generalizability of this study’s findings to clinical settings may be limited due to the use of an undiagnosed sample, which is often a limitation in the current literature (Botella et al., 2017; Garrett et al., 2018). The study would have benefited from more extensive assessment such as the Acrophobia Questionnaire (Cohen, 1977) or the Visual Height Intolerance Severity Scale (Huppert et al., 2017), and diagnostic interviews. The study could also have benefited from using physiological measures during testing. Considering we did not have any exclusion criteria beyond age, the inclusive sampling method may have benefited sample diversity. However, by employing convenience and snowball sampling, the sample was non-randomized and may have been biased toward more cooperative and interested participants and clinicians with positive inclinations toward VR and/or VRET. This may have resulted in a sample with more favorable attitudes than the general population of clinicians. Also, further studies are needed to determine the value of this solution for fear of heights in clinical samples using controlled designs. Further refining the scenarios could also be helpful. The lift scenario had the lift floating free in a street environment without any connection to relevant object such as a building or a construction vehicle. Further adjusting the scenarios could potentially increase the scenarios’ ecological validity. Another limitation is that the clinicians’ attitudes were not subjected to a systematic thematic analysis. The study-design did not allow for comparison of older vs. newer hardware. Therefore it is unknown whether the newer hardware is more effective, more immersive, or less associated with cybersickness. Future studies should take this into account and test for possible differences in treatment outcomes as well as differences in clinician’s attitudes toward newer and older hardware.

We propose that future work should investigate the clinical utility of up-to-date hardware and software. This may include testing if hardware with different features such as hand-tracking vs. controllers is associated with better treatment outcomes, as well as investigation of how different software may yield different treatment outcomes in clinical samples. Furthermore, the potential of VR as an authoring tool in clinical settings is a topic for further studies.

This study demonstrated that VRET for fear of heights can induce and reduce discomfort in a sample of clinicians and non-clinicians, and that clinicians’ attitudes toward VR in therapy become more positive after trying VRET for themselves. We suggest that dispelling negative beliefs among clinicians may increase the likelihood of wide-scale implementation of VR. Follow-up studies should further investigate if experience with VR dissuade concerns such as low realism/immersion and technological difficulties. Additionally, studies should further explore how newer VRET programs can be implemented and disseminated in clinical settings, and possibly as home-based training or self-help solutions.

## Data Availability Statement

The raw data supporting the conclusions of this article will be made available by the authors, without undue reservation.

## Ethics Statement

The studies involving human participants were reviewed and approved by Regional Committee for Medical and Health Research Ethics. The patients/participants provided their written informed consent to participate in this study. Written informed consent was obtained from the individual(s) for the publication of any potentially identifiable images or data included in this article.

## Author Contributions

ER, LH, and SS contributed to conception and design of the study. SS was the principal investigator. LH and ER were responsible for the data collection and wrote the first draft of the manuscript. All authors were responsible for the statistical analysis and contributed to manuscript revision, read, and approved the submitted version.

## Conflict of Interest

The authors declare that the research was conducted in the absence of any commercial or financial relationships that could be construed as a potential conflict of interest.
