# Supplementary material for: Virtual Reality Exposure Therapy for Fear of Heights: Clinicians’ Attitudes Become More Positive After Trying VRET
Source: Front Psychol. 2021 Jul 15;12:671871. doi: 10.3389/fpsyg.2021.671871 (PMC8319686; doi:10.3389/fpsyg.2021.671871)
Supplement: Supplementary file 1 [file Data_Sheet_1.docx]

**Appendix**

**Questionnaire (clinicians)**

**Pre-intervention**

| 1A | Age |  |
| --- | --- | --- |
| 1B | Sex |  |
| 1C | Years of work experience as a licensed clinician |  |
| 1D | Place of work |  |

| 2A | Have you tried VR (virtual reality) previously? | YES/NO |
| --- | --- | --- |
| 2B | Have you tried VR designed for mental health treatment previously? | YES/NO |
| 2Ca | Do you use exposure therapy in your practice? | YES/NO |
| 2Cb | (If YES on 2Ca) How many years of experience do you have? |  |

The next three questions are scored on a scale where ***0 is extremely negative***, 1 is very negative, 2 is quite negative, 3 is quite positive, 4 is very positive, and ***5 is extremely positive***. Please make a circle indicating your response.

| 3A | What is your general attitude towards novel technology? | 0 | 1 | 2 | 3 | 4 | 5 |
| --- | --- | --- | --- | --- | --- | --- | --- |
| 3B | What is your general attitude towards exposure therapy as a treatment method? | 0 | 1 | 2 | 3 | 4 | 5 |
| 3C | What is your general attitude towards using VR in therapy? | 0 | 1 | 2 | 3 | 4 | 5 |

The next four questions are scored on a scale where ***0 is not at all***, 1 is to a small extent, 2 is to some extent, 3 is to a moderate extent, 4 to a great extent, and ***5 to a very great extent***.

| 4A | To what extent do you think VR in treatment of phobia/anxiety may be useful? | 0 | 1 | 2 | 3 | 4 | 5 |
| --- | --- | --- | --- | --- | --- | --- | --- |
| 4B | To what extent do you think VR in treatment of phobia/anxiety may be a supplement to ordinary treatment? | 0 | 1 | 2 | 3 | 4 | 5 |
| 4C | To what extent do you think VR in treatment of phobia/anxiety may increase the feasibility of treatment? | 0 | 1 | 2 | 3 | 4 | 5 |
| 4D | To what extent would you say you are technologically literate? | 0 | 1 | 2 | 3 | 4 | 5 |

Please elaborate in the textboxes.

| 5A | What other advantages do you think VR in treatment may have? |
| --- | --- |
|  | |
| 5B | What disadvantages do you think VR in treatment may have? |
|  | |

On a scale from ***0 (no discomfort)*** to ***100 (extreme discomfort***).

| 6A | How would you rate your maximum level of discomfort when encountering heights? |  |
| --- | --- | --- |
| 6B | How would you rate your height related discomfort while on the footstool? |  |

**Post-intervention**

 On a scale from ***0 (no discomfort)*** to ***100 (extreme discomfort***).

| 7A | How would you rate your height related discomfort in VR? | SCENARIO 1  peak discomfort | SCENARIO 2  peak discomfort | SCENARIO 2  2 minutes after peak discomfort |
| --- | --- | --- | --- | --- |

The following questions are scored on a scale where ***0 is not at all***, 1 is to a small extent, 2 is to some extent, 3 is to some to a moderate extent, 4 to a great extent, and ***5 to a very great extent***.

| 8A | To what extent did you feel immersed in the VR-world? | 0 | 1 | 2 | 3 | 4 | 5 |
| --- | --- | --- | --- | --- | --- | --- | --- |
| 8B | To what extent did you perceive altitude? | 0 | 1 | 2 | 3 | 4 | 5 |
| 8C | To what extent do you think that VR in treatment of phobia / anxiety may be useful? | 0 | 1 | 2 | 3 | 4 | 5 |
| 8D | To what extent do you think that VR in treatment of phobia / anxiety may be a supplement to traditional treatment? | 0 | 1 | 2 | 3 | 4 | 5 |
| 8E | To what extent do you think VR in treatment of phobia/anxiety may increase the feasibility of treatment? | 0 | 1 | 2 | 3 | 4 | 5 |
| 8F | To what extent would you rate the likelihood that you will use VR in therapy in the future? | 0 | 1 | 2 | 3 | 4 | 5 |

The last question is scored on a scale where ***0 is extremely negative***, 1 is very negative, 2 is quite negative, 3 is quite positive, 4 is very positive, and ***5 is extremely positive***.

| 9A | What is your general attitude toward the use of VR in therapy? | 0 | 1 | 2 | 3 | 4 | 5 |
| --- | --- | --- | --- | --- | --- | --- | --- |

**Questionnaire (non-clinicians)**

**Pre-intervention**

| 1A | Age |  |
| --- | --- | --- |
| 1B | Sex |  |

The next question is scored on a scale where ***0 is extremely negative***, 1 is very negative, 2 is quite negative, 3 is quite positive, 4 is very positive, and ***5 is extremely positive***. Make a circle indicating your response.

| 2A | What is your general attitude toward novel technology? | 0 | 1 | 2 | 3 | 4 | 5 |
| --- | --- | --- | --- | --- | --- | --- | --- |

On a scale from ***0 (no discomfort)*** to ***100 (extreme discomfort***).

| 3A | How would you rate your maximum level of discomfort when encountering heights? |  |
| --- | --- | --- |
| 3B | How would you rate your height related discomfort while on the footstool? |  |

**Post-intervention**

The next two questions are scored on a scale where ***0 is not at all***, 1 is to a small extent, 2 is to some extent, 3 is to some to a moderate extent, 4 to a great extent, and ***5 to a very great extent***.

| 4A | To what extent did you feel immersed in the VR-world? | 0 | 1 | 2 | 3 | 4 | 5 |
| --- | --- | --- | --- | --- | --- | --- | --- |
| 4B | To what extent did you perceive altitude? | 0 | 1 | 2 | 3 | 4 | 5 |

On a scale from ***0 (no discomfort)*** to ***100 (extreme discomfort***).

| 5A | How would you rate your height related discomfort in VR? | SCENARIO 1 peak discomfort | SCENARIO 2  peak discomfort | SCENARIO 2  2 minutes after peak discomfort |
| --- | --- | --- | --- | --- |

*Note*. The questionnaires were originally in Norwegian but were translated for the purposes of this manuscript.
